# Temporal Delays Along the Neurosurgical Care Continuum for Traumatic Brain Injury Patients at a Tertiary Care Hospital in Kampala, Uganda

**DOI:** 10.1093/neuros/nyy004

**Published:** 2018-02-26

**Authors:** Silvia D Vaca, Benjamin J Kuo, Joao Ricardo Nickenig Vissoci, Catherine A Staton, Linda W Xu, Michael Muhumuza, Hussein Ssenyonjo, John Mukasa, Joel Kiryabwire, Henry E Rice, Gerald A Grant, Michael M Haglund

**Affiliations:** 1 Stanford University School of Medicine, Palo Alto, California; 2 Stanford Center for Innovation in Global Health, Palo Alto, California; 3 Duke University Division of Global Neurosurgery and Neurology, Durham, North Carolina; 4 Duke University Global Health Institute, Durham, North Carolina; 5 Duke-NUS Medical School, Singapore, Singapore; 6 Duke Emergency Medicine, Duke University Medical Center, Durham, North Carolina; 7 Department of Neurosurgery, Stanford University Medical Center, Palo Alto, California; 8 Department of Neurosurgery, Mulago Hospital, Kampala, Uganda; 9 Department of Surgery, Duke University Medical Center, Durham, North Carolina; 10 Department of Neurosurgery, Duke University Medical Center, Durham, North Carolina

**Keywords:** Global neurosurgery, Traumatic brain injury, Care continuum, Prospective registry, Mulago

## Abstract

**BACKGROUND:**

Significant care continuum delays between acute traumatic brain injury (TBI) and definitive surgery are associated with poor outcomes. Use of the “3 delays” model to evaluate TBI outcomes in low- and middle-income countries has not been performed.

**OBJECTIVE:**

To describe the care continuum, using the 3 delays framework, and its association with TBI patient outcomes in Kampala, Uganda.

**METHODS:**

Prospective data were collected for 563 TBI patients presenting to a tertiary hospital in Kampala from 1 June to 30 November 2016. Four time intervals were constructed along 5 time points: injury, hospital arrival, neurosurgical evaluation, computed tomography (CT) results, and definitive surgery. Time interval differences among mild, moderate, and severe TBI and their association with mortality were analyzed.

**RESULTS:**

Significant care continuum differences were observed for interval 3 (neurosurgical evaluation to CT result) and 4 (CT result to surgery) between severe TBI patients (7 h for interval 3 and 24 h for interval 4) and mild TBI patients (19 h for interval 3 and 96 h for interval 4). These postarrival delays were associated with mortality for mild (*P* = .05) and moderate TBI (*P* = .03) patients. Significant hospital arrival delays for moderate TBI patients were associated with mortality (*P* = .04).

**CONCLUSION:**

Delays for mild and moderate TBI patients were associated with mortality, suggesting that quality improvement interventions could target current triage practices. Future research should aim to understand the contributors to delays along the care continuum, opportunities for more effective resource allocation, and the need to improve prehospital logistical referral systems.

**ABBREVIATIONS**
CT: computed tomography
EDH: epidural hematoma
GCS: Glasgow coma score
HIC: high-income country
IQR: interquartile range
LMIC: low- and middle-income countries
OR: operating room
RA: research assistant
REDCap.: Research Electronic Data Capture
SDH: subdural hematoma
TBI: traumatic brain injury

According to The Brain Trauma Foundation, surgery to address traumatic brain injuries (TBI) including subdural hematomas (SDHs) and epidural hematomas (EDHs) should occur without delays for severe TBI patients, Glasgow coma score (GCS) < 9, with signs of brain herniation, and neurological deterioration.^1^ For severe TBI patients with acute SDH, the mortality rate for patients who received surgery within 4 h of injury in the United States was between 30% and 47% compared to 80% to 90% when surgery was delayed beyond 4 h.^2,3^ For severe TBI patients with EDH, the mortality rate increased from 17% to 65% when the duration between the onset of coma to surgical decompression was delayed beyond 2 h.^3^ In addition to the high mortality associated with an initial severe TBI presentation, the rate of neurological status deterioration throughout the hospital stay has also been highly predictive of poor outcomes.^4^ In a systematic review of studies on traumatic SDHs and EDHs with bilateral fixed and dilated pupils, despite the high mortality and morbidity, better functional recovery could be achieved in addition to higher survival rates by aggressive timely surgical interventions.^5^

In a recent study from Canada, a high-income country (HIC), temporal delays were analyzed in TBI patients and found that the median total time between emergency department arrival and operation room (OR) arrival was 150 min.^6^ The investigators identified potential bottlenecks at the computed tomography (CT) scanning and OR preparation stages that contributed to provider delays, which were found to be associated with poorer outcomes.

While delays to care exist in resource rich settings,^7^ relatively greater delays are seen along both the prehospital arrival and inter- and intrahospital continuums in low- and middle-income countries (LMICs) largely due to limited healthcare capacity to address the disproportional rates of TBI caused by road traffic injuries in Sub-Saharan Africa.^8,9^ A multitude of factors contribute to significant TBI surgical delays in LMICs. In a study looking at delays to surgical care in 21 LMICs, prehospital arrival delays could be explained by poor referral systems and geographical challenges while postarrival delays were mostly due to financial and infrastructural factors.^10^

While many LMICs have government subsidized systems to offset surgical costs, the burden of securing funds by the patients and their families for medications, supplies, and CT diagnostics poses a significant challenge to timely surgical interventions.^11,12^ In Kampala, Uganda, the challenge of obtaining timely CT scans is 2-fold. First, due to a lack of a functional CT scanner at the tertiary hospital, patients need to arrange their own transportation to the nearby private facility to obtain their CT scans. Second, because this is a private facility, patients need to pay for the CT testing, ranging from $80 to $130,^11^ which is near the average monthly income in Kampala.^13^

Initially coined in global maternal health to describe the contributors to maternal mortality, the “3 delays” model describes delays in (1) seeking, (2) reaching, and (3) receiving medical intervention.^14^ While there are increased studies examining obstetrical outcomes in LMICs using the 3 delays model,^15,16^ the use of this framework in analyzing TBI outcomes in LMICs has not been done. The objectives of this study are to (1) describe the temporal delays through a modified 3 delays model that fits the context of neurosurgical interventions for TBI patients in Kampala and (2) investigate the association between potential delays and mortality.

## METHODS

### Ethical Approval

Institutional review boards at the partner institutions in the United States and Uganda provided ethical approval for this study. Patient consent was not required for this secondary data analysis of a de-identified dataset.

### Study Design

A prospective registry of TBI patients was created using Research Electronic Data Capture (REDCap). All TBI patients presenting between 1 June 2016 and 30 November 2016 to the emergency department at a tertiary care hospital in Kampala and referred to the neurosurgery team were included in this study. This study is a secondary data analysis of this de-identified dataset for this 6-mo period. The registry data were collected over 70 h per week, 7 d a week by 2 trained Ugandan research nurses. The research assistants (RAs) observed patient care throughout their hospital stay, reviewed patient charts, collected data initially on paper forms, and entered all data into the REDCap registry following patient discharge. Quality analysis occurred at 2 stages: (1) during data collection by the local neurosurgery resident supervising the RAs to clarify clinical variables and check for accuracy, and (2) during data analysis by the local research program manager supervising the accuracy of data entry into REDCap. Data quality was assessed among the research team every 4 wk, and discrepancies were investigated by crosschecking paper collection forms as well as the original patient charts.

### Variables

The data collected included patient demographics, TBI severity on admission, management pathways, time intervals, and survival status. TBI severity was stratified by admission GCS level: mild (GCS 13-15), moderate (GCS 9-12), and severe (GCS 3-8). Management pathways were categorized into 4 groups: (1) surgery received, (2) surgery not received, (3) nonoperative, and (4) not admitted. The “surgery not received” group is a distinct cohort to designate patients admitted to receive surgical intervention, but who failed to receive timely surgical intervention due to preoperative death, patients and their families discharging against medical advice, and infrastructural limitations. Cases were only excluded due to missing interval times, constituting fewer than 5% of cases.

### Time Points and Intervals

**FIGURE 1.**
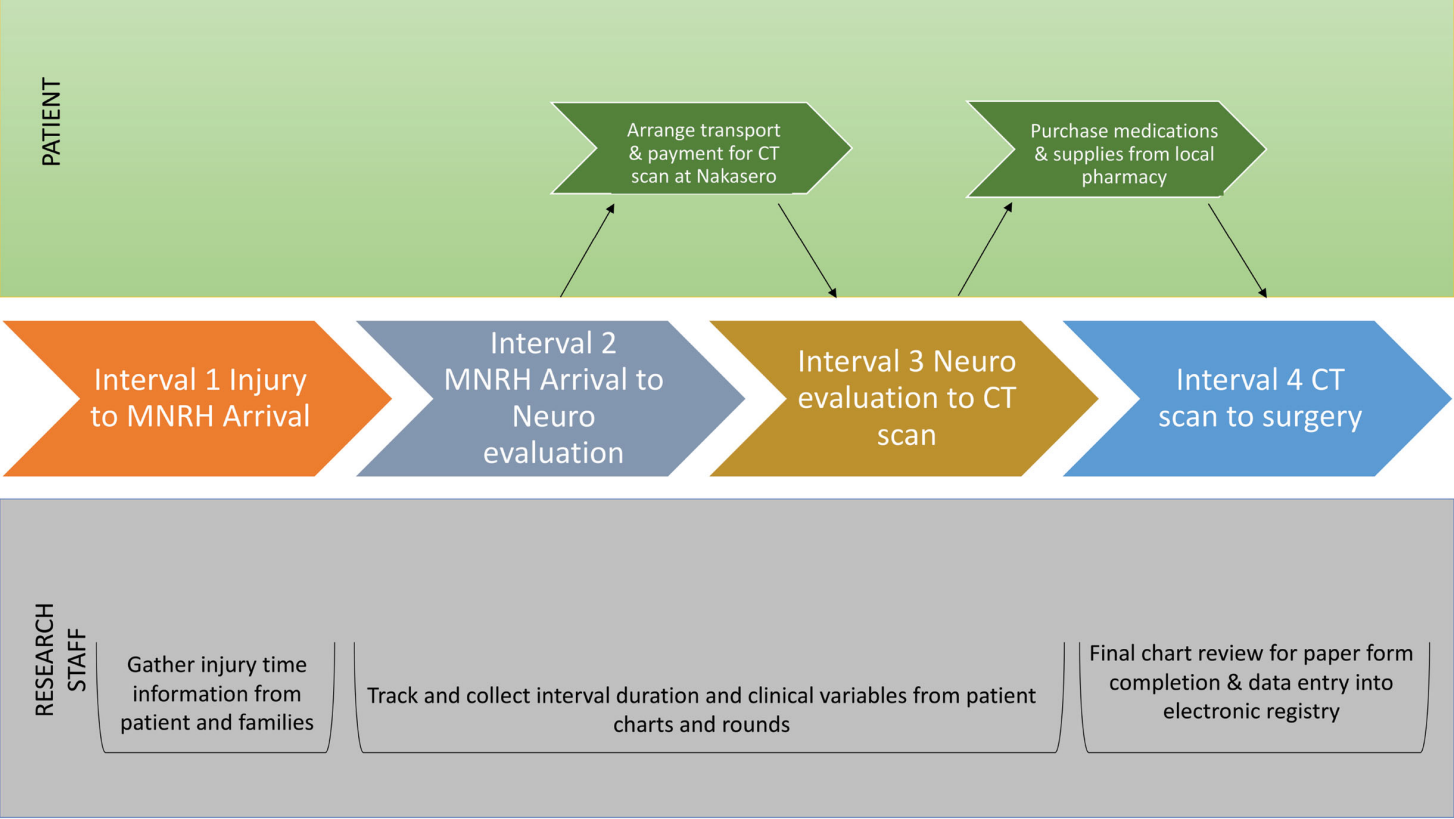
Study design workflow of the collection of neurosurgical care continuum variables using a modified “3 delays” modeled for traumatic brain injury.

Important neurosurgical continuum time points were determined to be (1) time of injury, (2) time of hospital arrival, (3) time of initial neurosurgery team evaluation, (4) time of CT scan availability from nearby hospital, and (5) time of surgery (Figure 1).

### Data Analysis

Statistical analyses were performed using Stata version 14.0 (Stata Corp, College Station, Texas). Descriptive statistics were calculated to describe patient characteristics and management pathways. Time intervals are reported in median and interquartile range and analyzed using the Kruskal-Wallis test given the nonparametric and positively skewed nature of the dataset. Univariate analysis was performed to examine differences in time intervals across TBI severity and survival status. Associations between interval delays and mortality were analyzed within each TBI severity group.

## RESULTS

### Patient Characteristics and Management Pathways

A total of 563 TBI patients were enrolled in this prospective study. More than 70% of the patients were aged 15 to 44 years and 86% were male (Table 1). The most common cause of TBI was road traffic injury (62%), and 242 patients (42%) were referred from primary care clinics.

**TABLE 1.** Patient Characteristics, Management Pathway, and Survival Status.

	Total cases (%)	Mild TBI (%)	Moderate TBI (%)	Severe TBI (%)
Total patients	563 (100.0%)	324 (100.0%)	152 (100.0%)	87 (100.0%)
**Demographics**				
Age (years)				
0-14	78 (13.8%)	40 (12.3%)	25 (16.5%)	13 (14.9%)
15-29	243 (43.4%)	146 (45.1%)	63 (41.4%)	34 (39.1%)
30-44	160 (28.3%)	95 (29.3%)	28 (18.4%)	27 (31.0%)
≥ 45	81 (14.3%)	43 (13.3%)	25 (16.4%)	13 (14.9%)
Male gender	488 (86.4%)	279 (86.1%)	134 (88.2%)	73 (83.9%)
Type of injury				
Assault	136 (24.4%)	89 (27.5%)	32 (21.1%)	15 (17.2%)
Fall	64 (11.3%)	34 (10.5%)	24 (15.8%)	6 (6.9%)
Road traffic injury	350 (62.0%)	192 (59.3%)	94 (61.8%)	64 (73.6%)
Occupation				
Unemployed	74 (13.1%)	40 (12.3%)	22 (14.5%)	12 (13.8%)
Self employed	180 (32.0%)	100 (30.9%)	49 (32.2%)	31 (35.6%)
Formal employment	58 (10.3%)	38 (11.7%)	13 (8.6%)	7 (8.0%)
Unknown	251 (44.6%)	146 (45.1%)	68 (44.7%)	37 (42.5%)
Primary care referral	242 (42.8%)	128 (39.5%)	74 (48.7%)	40 (46.0%)
CT done	440 (77.9%)	235 (72.5%)	132 (86.8%)	73 (83.9%)
Management pathway				
Surgery received	102 (18.1%)	63 (19.4%)	23 (15.1%)	16 (18.4%)
Surgery not received	29 (5.1%)	8 (2.5%)	10 (6.6%)	11 (12.6%)
Nonoperative	251 (44.6%)	128 (39.5%)	88 (57.9%)	35 (40.2%)
Not admitted	181 (32.2%)	125 (38.6%)	31 (20.4%)	25 (28.7%)
Mortality	54 (9.6%)	11 (3.4%)	21 (13.8%)	22 (25.3%)

The nonoperative cohort designates patients admitted for nonoperative management, while the surgery not received cohort represents a distinct group of patients admitted to receive surgical intervention, but failed to receive timely surgical intervention due to preoperative death, patients and their families discharging against medical advice, and infrastructural limitations. The top 3 CT diagnoses for the nonoperative cohort were contusions (n = 37, 15.5%), EDHs (n = 38, 15.9%), and more than one of the following without intracranial bleed: fracture, edema, or contusion (n = 39, 16.3%). For the surgery not received cohort, the top 3 CT diagnoses were acute SDH (n = 8, 29.6%), EDHs (n = 4, 14.8%), and more than one of the following without intracranial bleed: fracture, edema, or contusion (n = 6, 22.2%).

**FIGURE 2.**
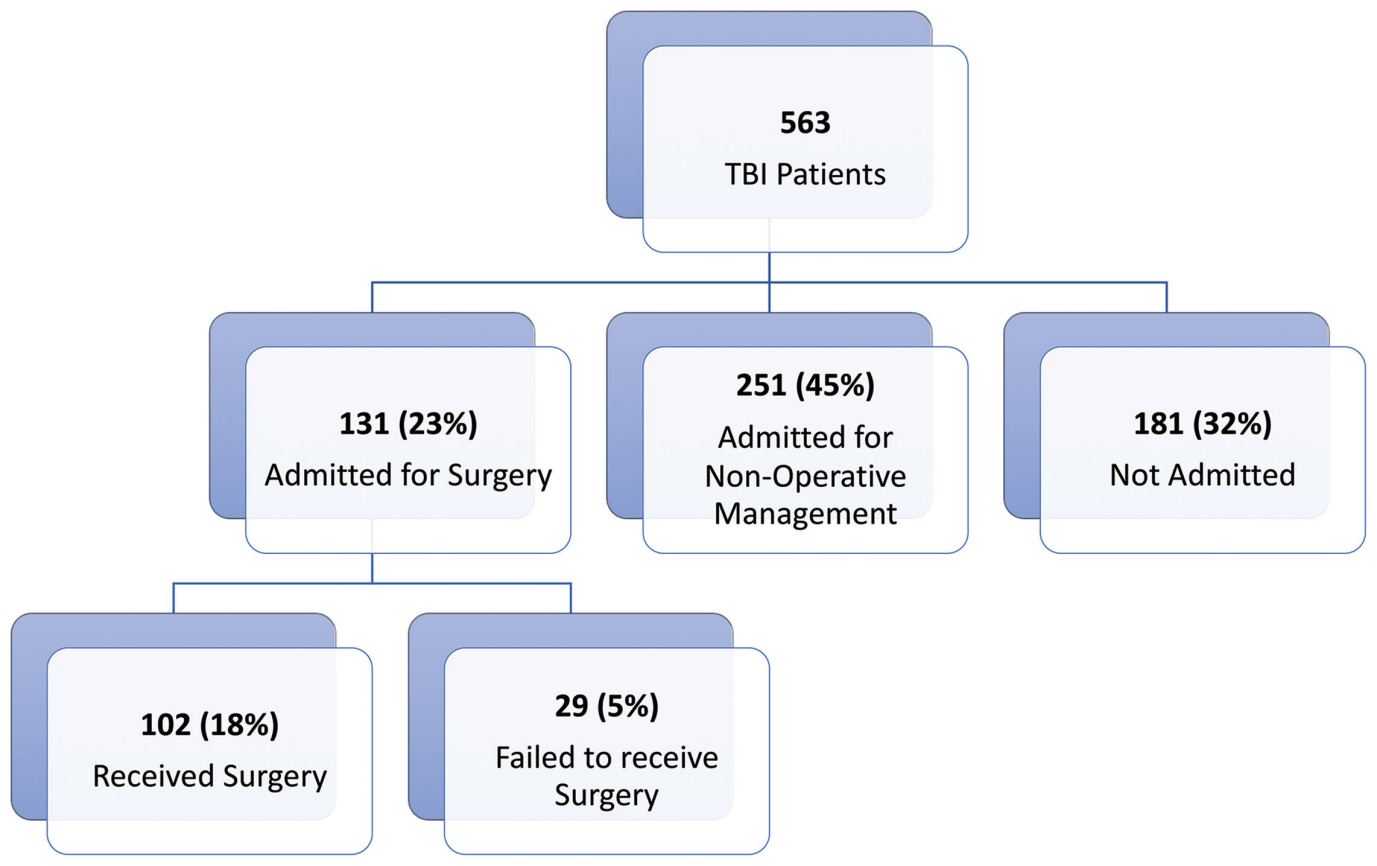
Management pathway of all patients.

Mild, moderate, and severe TBI represented 57.5%, 27.0%, and 15.5%, respectively (Table 1). CT scan results were available for nearly 78% of patients. Surgery was planned for 131 patients initially, but only 102 patients (18.1%) received it while 29 patients (5.1%) failed to receive timely surgery due to preoperative death, patients and their families self-discharging against medical advice, and infrastructural limitations (Figure 2). Nonoperative management was offered to 251 patients (44.6%) and 181 patients (32.2%) were not admitted. The “not admitted” patient group represented patients who were discharged directly from the emergency department by the neurosurgery team, did not return from the private hospital with CT scans, or decided to self-discharge against medical advice. Of the 25 severe TBI patients not admitted, 13 did not return with CT scans and the remainder did not have discharge data available.

### Distribution of Mortality Contribution by TBI Severity Across Management Pathways

**FIGURE 3.**
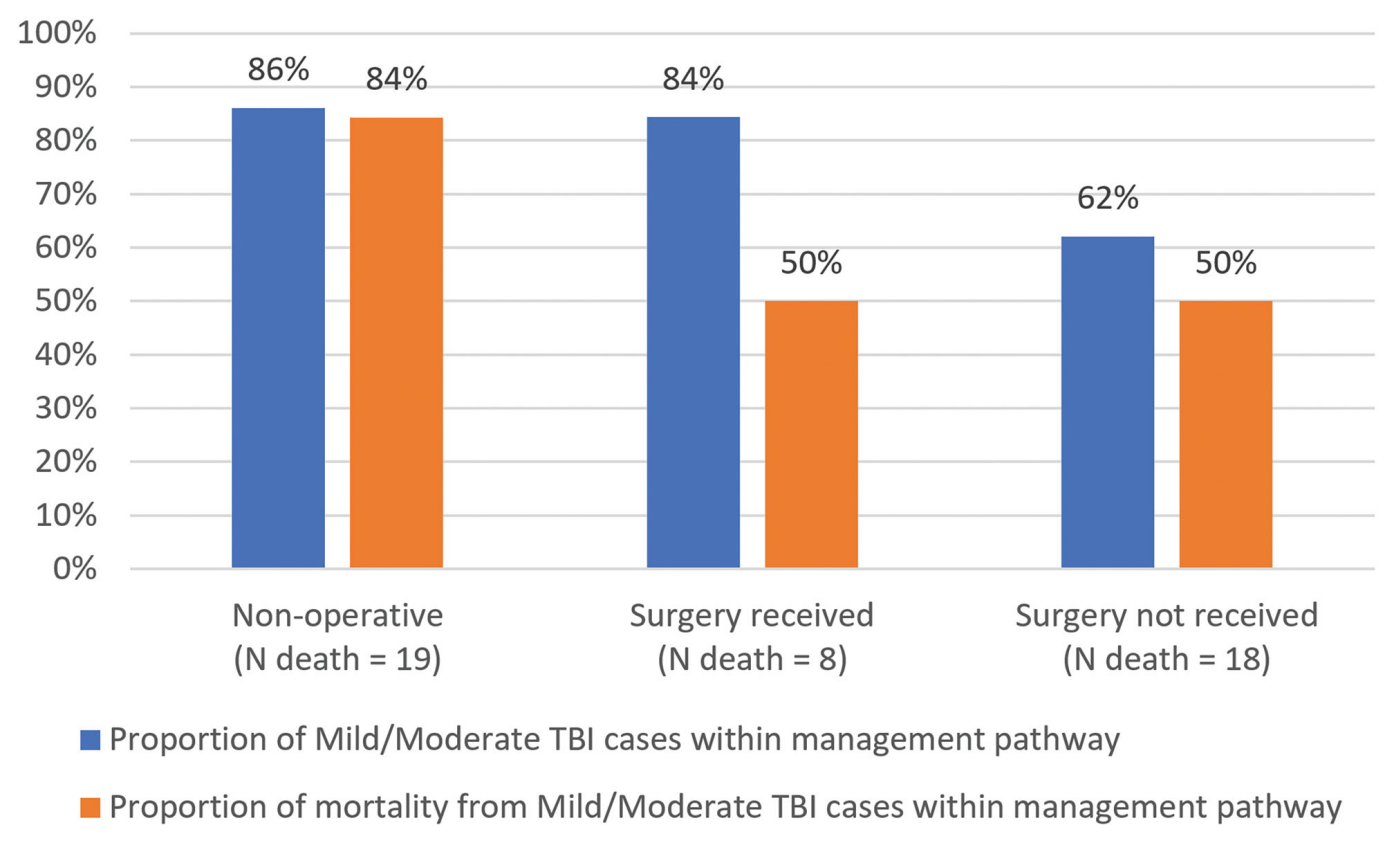
Relative contribution of mild and moderate TBI groups to mortality within management pathways. Compared to the surgery received group, the nonoperative group has a similar proportion of mild/moderate TBI cases but relatively higher mild/moderate TBI mortality. Likewise, compared to the surgery received group, the surgery-not-received group had a lower proportion of mild/moderate TBI cases, but equally high mild/moderate TBI mortality.

Mild and moderate TBI accounted for 50% of the mortality seen in both the surgery received (4 out of 8) and surgery not received cohorts (9 out of 18), despite the surgery-not-received cohort having a lower proportion of mild and moderate TBI cases (62% vs 84%). The proportion of mild and moderate TBI cases was similar between the nonoperative and surgery received cohorts (86 vs 84%), but the proportion of mild and moderate TBI mortality (16 out of 19) was higher (84% vs 50%) in the nonoperative cohort (Figure 3).

### Care Continuum Differences by TBI Severity

**FIGURE 4.**
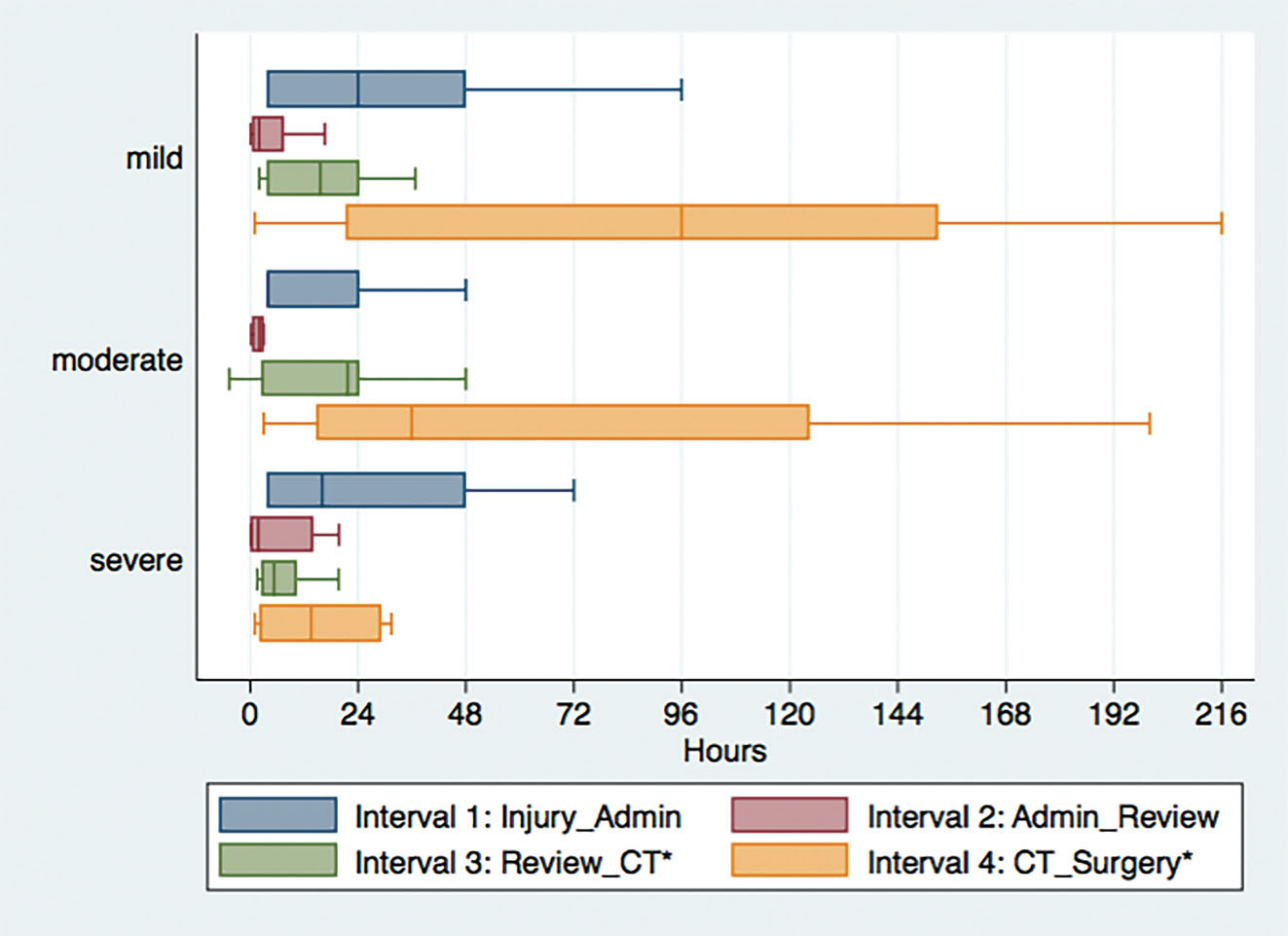
Time interval along the neurosurgical care continuum by TBI severity. *Interval 3 significantly differed by TBI severity (Kruskal-Wallis, *P* = .0193). *Interval 4 significantly differed by TBI severity (Kruskal-Wallis, *P* = .0435).

The median time from injury to hospital arrival (interval 1) was 4 h, from arrival to neurosurgery team evaluation (interval 2) was 2 h, from evaluation to CT results obtained from private hospital (interval 3) was 17 h, and from CT results to surgery (interval 4) was 92 h. Of the four time intervals, interval 4 had the greatest variability with an interquartile range (IQR) of 18-144 as compared to 4-24, 1-10, and 5-24 for intervals 1, 2, and 3, respectively. Intervals 3 and 4 varied significantly by TBI severity, *P*-values .0193 and .0435, respectively (Figure 4). Severe TBI patients had significantly shorter time intervals compared to both mild and moderate TBI patients suggesting that they received CT (7 vs 17 and 19 h) and surgery (20 vs 52 and 96 h) sooner. The median duration for the entire neurosurgical care continuum from injury to surgery was 129 h, and this was significantly different by TBI severity (*P* = .0048). Pairwise comparisons revealed significant differences across all three TBI severity groups (Table 2). The median injury to surgery durations were 174, 97, and 69 h for mild, moderate, and severe TBI, respectively.

**TABLE 2.** Pairwise Comparison of the Neurosurgical Care Continuum From Injury to Surgery by TBI Severity.

	Mild TBI	Moderate TBI	Severe TBI	
TBI severity group comparison	Median Hr (IQR)	Median Hr (IQR)	Median Hr (IQR)	*P*-value
Mild vs moderate	174 (120-306)	97 (55-263)		.0496
Mild vs severe	174 (120-306)		69 (26-89)	.0008
Moderate vs severe		97 (55-263)	69 (26-89)	.0500

*P*-value determined by the Kruskal-Wallis test.

### Care Continuum Association With Mortality

Stratifying by TBI severity, time interval 1 was significantly associated with mortality for moderate TBI patients (Table 3). Patients who died were more likely to have arrived later than those who survived (24 vs 4 h, *P* = .0491). Interval 2 was not associated with mortality within each TBI severity group. Delays within interval 3 were associated with mortality for mild (19 vs 24 h, *P* = .0500) and moderate (16 vs 24 h, *P* = .0335) TBI patients. While not significant, the reverse pattern was observed for severe TBI patients. Those who died received CT results (4 vs 8 h, *P* = .0527) and surgery (15 vs 26 h, *P* = .0571) sooner than those who survived. While interval 4 was noted to differ significantly across TBI severity groups, there was no association with mortality.

**TABLE 3.** Associations Between Time Interval and Mortality Stratified by TBI Severity.

	Mild TBI median Hr (IQR)			Moderate TBI median Hr (IQR)			Severe TBI median Hr (IQR)		
Care continuum time intervals	Alive	Died	*P* value	Alive	Died	*P* Value	Alive	Died	*P* value
Time interval 1 injury to arrival	4 (4-24)	4 (4-24)	.89	4 (4-27)	24 (4-120)	**.04**	4 (4-24)	4 (4-24)	.53
Time interval 2 arrival to review	2 (1-9)	3 (0-11)	.92	2 (1-11)	3 (2-16)	.12	1 (0-13)	1 (0-1)	.19
Time interval 3 review to CT	19 (6-24)	24 (16-108)	**.05**	16 (6-24)	24 (4-48)	**.03**	8 (4-24)	4 (3-11)	**.05**
Time interval 4 CT to surgery	96 (48-147)	NA	NA	52 (18-96)	64 (3-124)	.59	26 (7-120)	15 (3-59)	.06
Total arrival to surgery	126 (74-191)	NA	NA	69 (32-230)	84 (8-160)	.59	68 (23-244)	19 (7-65)	.15
Total injury to surgery	174 (120-306)	NA	NA	97 (56-263)	100 (12-164)	.42	71 (43-89)	25 (13-91)	.18

*P*-value determined by the Kruskal-Wallis test.

## DISCUSSION

### Summary

To our knowledge, this is the first analysis using a modified “3 delays” framework to analyze the time continuum of TBI patients in Uganda from injury to surgery. Substantial delays were identified at every step of the care continuum. Initial findings showed that most mild and moderate TBI deaths came from the nonoperative cohort of patients not receiving surgery. We found significant associations between delays along the care continuum and mortality for mild and moderate TBI patients suggesting a potential opportunity for optimizing resource allocation to improve TBI outcomes.

### The Need to Account for Care Continuum Delays

The overall TBI mortality rate of 9.6% is comparable to studies in HICs^17,18^ and while the impact of intensive care unit entry and ventilator support have been shown to substantially worsen outcome,^19,20^ less is known about the impact of various delays in seeking, reaching, and receiving care. A study of TBI outcomes in Tanzania found a comparable overall mortality rate, but saw mortality increase dramatically to 48% for severe TBI patients,^21^ which was largely associated with delayed arrivals compared to severe TBI mortality rates of 28% seen in high-income settings.^22^ The challenges of timely hospital arrival in LMICs are multifaceted and have been previously documented, which include self-treatment practices, primary clinic visits, and poor referral systems.^16,23,24^ The time intervals varied across TBI severity. Therefore, to fully understand the impact of delays along the care continuum for TBI patients in LMICs, more granularity is needed in future studies. This detail would more clearly reveal patterns in arrival time influenced by not only TBI severity but also geographical distribution and socioeconomic status. Along the postarrival spectrum, stratifying time continuum intervals by TBI severity, management pathways, and other infrastructural limitations would better reveal relationships among factors that influence delays to providing care.

### Shunting of Resources to the Neediest

As previously noted, while severe TBI patients experienced considerable delays from injury to surgery (69 h), they had a significantly shorter time continuum from injury to surgery as compared to both mild (174 h, *P* = .0008) and moderate (97 h, *P* = .0500) TBI patients. This indicates that sicker patients tend to seek, reach, and receive care sooner than patients with milder conditions, suggesting a current neurosurgical triaging practice that arranges for CT scans and provides surgical interventions to the neediest patients first. Furthermore, extended delays along time intervals 3 (neurosurgical evaluation to CT results) and 4 (CT results to surgery) were significantly associated with mortality for both mild and moderate TBI patients. This overall pattern suggests that there are greater delays experienced by patients presenting initially with milder conditions due to the shunting of resources to address the neediest patients first.

### Extended Delays to Surgical Care

Extended delays between injury and surgical interventions for TBI are highly correlated with poor outcomes. In a sample of 82 comatose patients in HIC, 1 study showed mortality tripling from 30% to 90% if surgery took place more than 4 h after injury.^2^ In another study of 171 severe TBI patients, mortality more than doubled and tripled to 80% and 65% for SDH and EDH, respectively.^3^ While the postoperative mortality rate for all TBI reported here is 7.8%, this increased dramatically to 62% for patients who were admitted for surgery but did not receive it. Within this group, mortality further increased to 81.8% for severe TBI patients. Moreover, this in-hospital mortality rate does not capture a large proportion of patients who died prior to reaching the hospital and instead are captured by the Kampala City Council Mortuary.^25^ Contributing to the arrival delay is the inadequate prehospital support system by first responders who lack proper supplies and training to provide appropriate initial trauma support.^26^

Prior to surgery, patients will need to have CT scans done outside of the tertiary care hospital, which requires the patients to first be stabilized and transport services arranged. Reported contributors to this delay are (1) the need for patients and their families to arrange their own transportation to the nearby private facility which had the only functional CT scanner during this study period; (2) the need for families to come up with the funds to pay for CT testing, which is approximately 250 000 shillings—almost the same as the average monthly income in Uganda; and (3) the need for severe TBI patients to be stabilized prior to getting CT testing.^13^ This delay could be markedly decreased with the ownership and maintenance of a functional CT scanner at the tertiary care hospital. While this facility owns a CT scanner, it was not functional during the study time period and the single biomedical engineer employed by the hospital was unable to repair it. The work presented here stresses the importance of timely and affordable CT imaging to the management of TBI patients. A cost-effectiveness analysis would further strengthen the argument for this expensive equipment and skilled human capital in a public facility with limited funding.

Following CT results, it takes nearly 4 d (92 h) for most patients to receive surgery. This duration was especially extended for mild (96 h) and moderate (52 h) TBI patients compared to severe (20 h) TBI patients. Major contributors to delays along this interval are theatre space limitations and the extended time managing complications prior to surgery. During the study time period, only 1 OR was dedicated to neurosurgical care. The number of cases performed daily was additionally constrained by the availability of anesthesia staff and sterilized surgical equipment. With a perpetual imbalance between surgical demand and surgical capacity, the most emergent cases were performed first and those with milder presentations were further delayed.

In a setting where delays to surgery are an order of magnitude longer, it is difficult to translate conclusions from HIC studies regarding the benefit of minutes to hours saved. At extended time points, it is likely that patients presenting to the hospital have already undergone a more extensive natural triage than the patients reflected in the HIC studies. For these patients, stabilization at smaller hospitals, clinics, or basic emergency services may play a key role in increasing access to surgical care.^27^ Continued research in LMICs is needed to elucidate the association between surgical delays and patient outcomes at extended time points. Furthermore, the stark delays at every interval along the care continuum stress the need for not only operative capacity building, but also consideration of intrahospital and prehospital barriers to surgery.

### Opportunities to Increase Surgical Capacity and Optimize Resource Allocation

Currently, mild and moderate TBI patients account for a substantially higher proportion of the nonoperative mortality than surgical mortality (84% vs 50%), even though the proportion of mild and moderate TBI cases in these cohorts (84% vs 86%) is comparable. Similarly, despite a lower proportion of mild and moderate TBI cases (62%) in the surgery-not-received group, mild and moderate TBI patients still account for 50% of the mortality in this cohort. Several factors may have contributed to the increased mortality observed in the mild and moderate TBI cohorts, including the need for early diagnosis and timely intervention—through either surgery, intensive care, emergency care, or early detection. Therefore, it is essential for future studies and interventions to assess this increased mortality through the broader context of the entire medical system, including full nonsurgical medical care as indicated.

The finding that resources are potentially being shunted to sicker patients resulting in extended delays along the continuum for mild and moderate TBI patients coupled with the association between these delays and increased mortality highlights a potential role for optimizing resource allocation to improve outcomes. Further research to identify (1) specific factors influencing delays in these patient groups and (2) the current determinants for neurosurgical interventions would help elucidate the interplay of clinical variables and time continuum intervals and how they affect TBI outcomes. This is needed to more effectively streamline services to patients with the greatest needs and best survival odds and to complement existing neurosurgical education programs. Additionally, future studies should not only assess in-hospital mortality but also postdischarge neurological outcomes to better characterize the quality of life impact of neurosurgery across TBI severity groups.

### Limitations

This study was largely limited by the ability to collect accurate and complete continuum variables from patients and their families. The most challenging aspect was collecting accurate injury to arrival data on severe TBI patients who could not accurately respond and whose family members were not present. These circumstances were noted and extensive efforts were made to contact families and speak to the triage team to gather as much information as possible about the timing of injury. Similarly, incomplete operative variables limited our analysis. Of the 102 surgical patients, operation type was collected for only 40 patients: 16 burr holes, 15 craniectomies, and 9 craniotomies. For small cohorts, such as the mild TBI surgical deaths (n = 2), missing surgery dates prevented injury to surgery time calculations. These data limitations create the potential for confounding effects on mortality. With more complete data in a larger sample size, future analyses may refine and expand on the findings presented here. As is often the case in LMIC studies, this limitation underscores the need for improved clinical documentation both for facilitating patient care and research to improve patient outcomes.^23,28,29^ Through our continued partnership, we have improved documentation and supported capacity building for long-term data collection such that future studies may draw upon more extensive data.

## CONCLUSION

While the overall 9.6% mortality rate of all TBI patients presenting to this hospital may be comparable to high-income settings, this rate ranged from 4.7% for mild and moderate TBI patients receiving surgery to 81.8% for severe TBI patients who failed to receive surgery. The duration from injury to surgery varied considerably across TBI severity with the largest gap seen between mild TBI (174 h) and severe TBI (69 h) patients. Further stratification revealed significant associations between delays along the neurosurgical care continuum and mortality particularly for mild and moderate TBI patients, which underscores opportunities to optimize resource allocation for the mild and moderate TBI patients currently managed nonoperatively or failing to receive timely surgery. Further research is needed to determine factors contributing to delays along the care continuum for specific patient groups and whether improved timely interventions and increased surgical capacity could better improve TBI outcomes in Uganda.

### Disclosures

Funding was provided by the Duke Department of Neurosurgery, Duke Division of Global Neurosurgery and Neurology, Duke Global Health Institute, Duke-NUS Medical School, and Stanford Medical Scholars Program. The authors have no personal, financial, or institutional interest in any of the drugs, materials, or devices described in this article.

## Notes

The following abstract was submitted and accepted for publication for the 20th International Conference on Global Health and Innovation, April 24-25, 2018, entitled “Temporal Delays along the Neurosurgical Continuum for Traumatic Brain Injury Patients in Mulago Hospital in Kampala Uganda”, with authors Vaca, Kuo, Vissoci et al.

## COMMENT

This is a very important topic and it is very important that it be studied. It allows us to look at several important facets including the fact that thoughtful research can be done in low-income situations and under poor conditions, that we can learn tremendously from those situations - lessons which are applicable both to the developing world, and the high-income areas that we presently work in. It is, however, a thought provoking paper of what we must do and what we can accomplish in areas of limited reserves. It also shows that data must be interpreted most carefully.

In this article the authors looked at a 6-month period of time for head trauma patients coming to the Kampala Hospital. These 563 people were studied and they looked at delays in care or duration of care, dividing it into intervals on a model of usual delays, those which might take place prior to arriving to the hospital. This would tell about infrastructure, transportation, local practices, and recognition of injury; those which take place from time of arrival to time of neurosurgical consultation, and time for neurosurgical consultation to achieving the results of a CT scan, and the time from the CT scan to surgery if applicable. They discovered that a significant number of patients were not planned for operative treatment, which is reasonable, but they noticed also a rather high mortality rate. The data suggests that although the mortality for severe injury was as expected, it was relatively high for that of the mild and moderate head injury patients and that these patients had a considerably longer delay to diagnosis and initiation of surgical treatment than those in the severe case. The implications of this data are very important but must be interpreted very carefully. First of all, it suggests that triage is absolutely essential. Triage is not necessarily based on the most severe, but is often based on those that can benefit the most from the treatments which are available when one has limited resources. This is a very important concept that the authors express. The data, however, does need further explanation. There were several important lessons to be drawn from this paper.

1) We have a lot of work to do. If the time to care is measured in 124 hours, we have significant pre-hospital, intra-hospital, and OR capacity issues to address. This is an obvious situation and it is important to document exactly so we can work to improve and follow up on this.

2) If we are dealing with patients who primarily are coming in terms of days instead of hours after their injury, all of our data from high-income countries (which suggest improvements measured in hours gained or even minutes gained in care) may not be relevant as a natural triage may have already taken place with these people to a greater extent in the lower income countries so that they do not appear pre-hospital - ever, or quite incapacitated.

3) The next important issue is the interpretation of their data regarding nonsurgical care and those who did not receive surgery. This data needs to be explained further because it is very important. If the death rates are increased in the patients who did not receive surgery, one must either realize that the intensive medical care and early diagnosis may be more important than surgery in these people with the less severe initial presentations, so this puts responsibility on the entire medical system, not simply surgery alone. And, this also calls the questions whether the surgery may have been a more appropriate initial treatment recommendation in those patients. We do not have data to answer that question here, but further studies must take into account the logical conclusion that more of the mild and moderates should be salvaged by applying full treatments, both medical and surgical.

**Robert J. Dempsey**

*Madison, Wisconsin*
